# Detection of clonal antigen receptor gene rearrangement in dogs with lymphoma by real-time polymerase chain reaction and melting curve analysis

**DOI:** 10.1186/1746-6148-10-1

**Published:** 2014-01-03

**Authors:** Kathrin FA Langner, Alexa E Joetzke, Verena Nerschbach, Nina Eberle, Hans-Joachim Schuberth, Mirja Koy, Ingo Nolte, Daniela Betz

**Affiliations:** 1 Small Animal Clinic, University of Veterinary Medicine, Buenteweg 9, 30559 Hannover, Germany; 2 Immunology Unit, University of Veterinary Medicine, Bischofsholer Damm 15, 30173 Hannover, Germany

**Keywords:** Lymphoma, Dog, Real-time polymerase chain reaction, Melting curve analysis

## Abstract

**Background:**

Molecular techniques that detect canine lymphoma cells by their clonal antigen receptor gene rearrangement play an increasing role for diagnosis as well as for monitoring minimal residual disease during and after cytostatic therapy. However, the methods currently available are time-consuming and/or cost-intensive thus impeding the use in clinical routine. The aim of the present study was to develop and evaluate a real-time polymerase chain reaction (PCR) with subsequent melting curve analysis (MCA) for the detection of clonally rearranged antigen receptor genes in dogs with B and T cell lymphoma on non formalin-fixed and paraffin-embedded lymph node samples.

**Results:**

In lymph node aspirates from 30 dogs with multicentric B cell lymphoma, real-time PCR with MCA detected clonal rearrangement in 100% and conventional PCR with polyacrylamide gel electrophoresis (PAGE) in 93% of samples. Both methods correctly identified clonality in 80% of lymph node aspirates of 10 dogs with T cell lymphoma. None of the two PCR systems detected clonal rearrangement in samples from 9 dogs with lymph node hyperplasia. Using a dilutional series with regular lymphoid desoxyribonucleic acid (DNA), detection limits of lymphoma DNA were as low as 0.8% and 6.25% for B and T cell clonal rearrangement with real-time PCR and MCA and at 3.13% and 12.5% with the conventional system. Median absolute detection limits of lymphoma DNA were shown to be at 0.1 ng and 1 ng for the B and T cell immunophenotype with the real-time PCR system and at 10 ng each with conventional PCR and PAGE.

**Conclusions:**

Real-time PCR with MCA is a convenient and reliable method with a good analytical sensitivity. Thus, the method may assist the detection of clonal antigen receptor gene rearrangement in canine lymphoma patients in a clinical setting also in the presence of small amounts of neoplastic cells.

## Background

Lymphoma is one of the most common hematopoetic malignancies in dogs [1,2]. Detection of large amounts of tumor cells is reliably achieved by standard diagnostic methods such as cytology and flow cytometry [3-5]. The assessment of small cell numbers, however, requires highly sensitive molecular-based assays that detect lymphoma cells by their clonal rearrangement of the antigen receptor [6]. Within the past years, polymerase chain reaction (PCR) techniques have been successfully established for molecular staging of lymphoma patients as well as for the evaluation of minimal residual disease (MRD) during and after chemotherapy [7-10].

Current PCR strategies in canine lymphoma include a conventional system that utilizes universal primers for the amplification of the clonally rearranged third complementarity determining region (CDR3) of the antigen receptor and subsequent polyacrylamide gel electrophoresis (PAGE) for the analysis of PCR products [6,7]. Moreover, a probe-based real-time PCR assay has been developed using allele-specific primers, probes and standard curves, enabling a highly sensitive detection of canine lymphoma cells by their individually rearranged CDR3 [8]. Despite their sensitivity, both assays impede the widespread use in clinical routine: Conventional PCR systems with PAGE are time-consuming and have a risk of carry-over contamination due to the need of post-PCR processing of samples. Recent use of capillary electrophoresis instead of PAGE circumvented these issues [11,12] but the method is not yet standard equipment in veterinary research. Probe-based real-time PCR requires high technical experience as well as a labour- and cost-intensive individual set-up with CDR3 specific primers for each patient.

Real-time PCR methods with subsequent melting curve analysis (MCA) using universal primers and fluorescent dyes have been successfully applied in malignant lymphoproliferative neoplasias in humans such as acute and chronic lymphocytic leukaemia, non-Hodgkin’s lymphoma and cutaneous T cell lymphoma [13,14]. The assays have been shown to be both specific and sensitive for diagnostic purposes as well as for monitoring MRD. Following the amplification reaction, MCA is carried out in the same vessel by gradually raising the temperature. Specific melting temperatures of the amplicons (T_m_), determined by their individual length, sequence, G:C content and Watson-Crick base pairing are displayed as peaks by plotting the negative first derivative of the fluorescence versus the temperature [15]. Clonal samples containing large amounts of a single PCR product appear as tall, distinct and symmetrical peaks on MCA, whereas non-neoplastic lymphoid samples, resembling small amounts of several different amplicons, present as flat, wide and/or irregular peaks.

Therefore, we hypothesized that real-time PCR with MCA would be a specific and sensitive method to obtain molecular data for canine lymphoma patients in an economical way. In the current study, we evaluated a newly developed real-time PCR system with MCA in comparison to a conventional PCR method with PAGE for the detection of clonal antigen receptor gene rearrangement in lymph node aspirates of dogs with B and T cell lymphoma.

## Methods

### Animals

Forty dogs presented for clinically and cytologically confirmed multicentric lymphoma at the Small Animal Clinic, University of Veterinary Medicine Hannover, Germany, between 2008 and 2012 were enrolled in the study. Further inclusion criteria were clinical staging according to the World Health Organization staging system for domestic animals [16] with thoracic and abdominal radiographs, abdominal ultrasound, cytological assessment of aspirates from liver, spleen and bone marrow. Moreover, determination of the immunophenotype and application of combination chemotherapy [17] were required for enrollment. Patients that had received cytostatic therapy or immunosuppressant drugs for any reason were excluded from the study. Seventeen of the dogs were females (10 spayed) and 23 were males (8 castrated). Dogs were between 1 and 16 years (mean 7 years). The following breeds were included: mixed breed (7), Bernese Mountain Dog (6), Rottweiler (4), Cocker Spaniel (2), Small Munsterlander (2), Labrador Retriever (2), Golden Retriever (2), Boxer (2), Beagle (1), Norwich Terrier (1), Pug (1), Bullmastiff (1), Border Collie (1), Dachshound (1), Hovavart (1), Miniature Schnauzer (1), English Setter (1), Shetland Sheepdog (1), Australian Shepard (1), Old German Shepard (1) and West Highland White Terrier (1).

Six of the dogs were classified as clinical stage III, 21 dogs as stage IV and 13 dogs as stage V. Fifteen dogs had substage a and 25 dogs had substage b. Immunophenotyping using flow cytometric analysis of lymph node aspirates was performed as described previously [18] and revealed lymphoma of the B and T cell phenotype in 30 and 10 cases, respectively.

Lymph node samples of 9 dogs with cytologically confirmed lymph node hyperplasia served as the polyclonal control. A total of 12 lymph node samples containing regular lymphoid tissue was obtained from freshly euthanized dogs and was used for the polyclonal control with real-time PCR as well as for the dilutional series of lymphoma DNA with regular lymphoid DNA.

The study design was reviewed and approved by the Ethics Committee for Animal Experiments of Lower Saxony, Germany (33.9-42502-05-07A512). Written owner consent was obtained for all client-owned dogs prior to study enrollment.

### Sample preparation

Lymph node aspirates or biopsies were obtained from dogs with and without lymphoma and stored at -70°C until used for desoxyribonucleic acid (DNA) extraction. Sampling sites included the cervical, popliteal or sternal lymph nodes. A total of 2 samples were taken per site. The first sample was used for cytology and flow cytometry (needle flushed with medium to obtain remaining cells). The second sample was subjected to DNA extraction using the DNeasy extraction kit (Qiagen, Hilden, Germany) according to the manufacturer’s instructions. Concentration and purity of DNA preparations was determined on a spectrophotometer (Eppendorf, Hamburg, Germany).

### Conventional PCR and PAGE

Polymerase chain reaction was performed on lymphoid DNA using 2 sets of primers for the assessment of clonal rearrangement of the immunoglobuline heavy chain (IgH major and minor, B cell lymphoma) and 1 set for the detection of rearranged T cell receptor γ locus (TCRγ, T cell lymphoma) as previously described [6]. Amplification of the constant region gene of immunoglobuline M (Cμ) was performed on all samples to ensure quality of lymphoid DNA. All samples were analyzed in duplicates with all 4 sets of primers including a non-template control for each run. Briefly, samples were heated for 15 min at 94°C followed by 35 cycles of 8 sec at 94°C, 10 sec at 60°C and 15 sec at 72°C. Initially, a total of 100 ng of DNA were used per reaction. If no band was detected, an increased amount of 500 ng of DNA was used. Amplicons were subsequently subjected to PAGE on 12.5% native polyacrylamide gels. Gels were stained with GelRed (Biotium, Hayward, CA), and bands were visualized on a Bio-Vision imaging system (Vilber Lourmat, Marne La Vallee, France). Evaluation for clonal antigen receptor gene rearrangement was done as previously described in dogs with lymphoma [6]. Briefly, single or few distinct bands were indicative for clonal antigen receptor gene rearrangement whereas a smear or several faint bands were consistent with the absence of lymphoma cells in the sample. Abscence of pseudoclonality was determined by similar size of bands on duplicate samples.

### Real-time PCR and MCA

Amplification and subsequent MCA were run in 96-well optical reaction plates (Applied Biosystems, Darmstadt, Germany) using a StepOnePlus amplification and detection system (Applied Biosystems). A standard reaction mixture contained 100 ng of sample DNA, one set of primers and the SYBR Green I PCR master mix (Applied Biosystems) at a total volume of 25 μl. All samples were analyzed with all 4 primer sets. Samples were run in triplicates to reduce risk of false positive or false negative results due to technical errors. Pseudoclonality was ruled out by identical T_m_ and superimposable peaks on triplicate analysis. A total of 100 ng of sample DNA was used per reaction. For each run a clonal control consisting of lymphoma DNA, a polyclonal control of regular lymphoid DNA, and a non-template control were included. Preincubation was performed for 10 min at 95°C followed by 30–60 cycles of 15 sec at 95°C for denaturation and 45 sec at 60°C for annealing and extension. Fluorescence was measured at the end of each cycle. After completion of amplification, MCA was performed by gradually raising the temperature by 0.3°C/sec to 95°C during continous fluorescence monitoring. Negative first derivative of the fluorescence was plotted versus the temperature at the individual T_m_ of the amplicon to obtain easily assessable melting peaks [15]. All fluorescent reporter signals were measured against the internal reference dye (ROX) signal to normalize for non-PCR related fluorescence fluctuations between wells. Data were analyzed using the StepOne v2.0 software (Applied Biosystems). Evaluation of clonal rearrangement was done as previously described for human lymphoproliferative neoplasias [13,19] and was represented by a distinct symmetrical peak on MCA whereas regular lymphoid or hyperplastic tissue appeared as flat, irregular and/or wide peaks. To establish a reliable diagnosis of clonality, different primer concentrations using lymphoma DNA and non-template control were tested according to the manufacturer’s instructions. Finally, the peak height (-d[F1]/dT) ratio between sample and regular lymphoid DNA was optimized by adjusting the starting point of MCA and the number of amplification cycles [20-22].

### Determination of detection limit

Logarithmic serial dilutions of lymphoma samples with water (100 to 0.01 ng) and 2-fold dilutional series with regular lymphoid tissue (50% to ~0.4% of lymphoma DNA) were subjected to analysis by conventional and real-time PCR. Depending on the previously determined type of rearrangement, corresponding primer sets for either B or T cell analysis were chosen. Samples were run in duplicates with conventional PCR and in triplicates with real-time PCR for each dilutional step. The regular lymphoid DNA used for the dilution was a mix of 12 samples of the 3 dogs without cytological lymph node abnormalities. The mixture was prepared to adjust for potential variations between individual samples. Limit of detection was defined as the last concentration producing a visible band on PAGE or distinct peak with MCA.

### Statistics

Specificity and sensitivity for real-time PCR with MCA in comparison to the conventional PCR system were calculated according to the method of Gerstman and Cappucci [23].

## Results

### Establishment of real-time PCR with MCA

**Figure 1:**
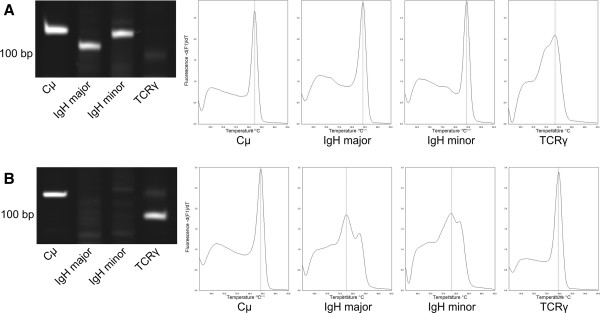
**Evaluation of clonal rearrangement of canine lymphoma samples by PAGE and MCA.** Samples of dogs with B cell lymphoma **(A)** and T cell lymphoma **(B)** were amplified with 4 primer sets for lymphoid DNA control (Cμ), B cell clonality (IgH major and minor) and T cell clonality (TCRγ) by conventional and SYBR Green I real-time PCR. Clonal samples concordantly revealed single sharp bands and distinct tall peaks on subsequent PAGE and MCA.

Real-time PCR carried out with the same primer concentrations and numbers of amplification cycles as conventional PCR revealed a single sharp peak in all samples using the Cμ primer set whereas samples from dogs with B and T cell lymphoma had distinct large peaks with the corresponding primer sets IgH major, minor and TCRγ (Figure 1). The amplification of regular lymphoid DNA showed irregular peaks of smaller sizes. With both real-time PCR and conventional PCR the same loci were found clonally rearranged.

In a first optimization step, primer concentrations revealing the highest signal intensity were identified (Table 1).

**Table 1:** Real-time PCR reaction conditions

**Product**	**Primer**		**Amplification conditions**	
	Name*	**Amount per reaction (nM)**	**Annealing (°C)**	**Number of cycles**
Cμ	Sigmf1	120	60	40
	Srμ3	120		
IgH major	CB1	120	60	50
	CB2	120		
IgH minor	CB1	120	60	40
	CB3	360		
TCRγ	TCRγ1	60	60	40
	TCRγ2	60		
	TCRγ3	360		

*Primer names and sequences according to Burnett et al. [6].

Subsequently, starting temperature of MCA was set at 78°C for IgH major and minor and at 75°C for TCRγ as it reduced analysis time and peak height of the polyclonal control compared to the clonally rearranged samples.

**Figure 2:**
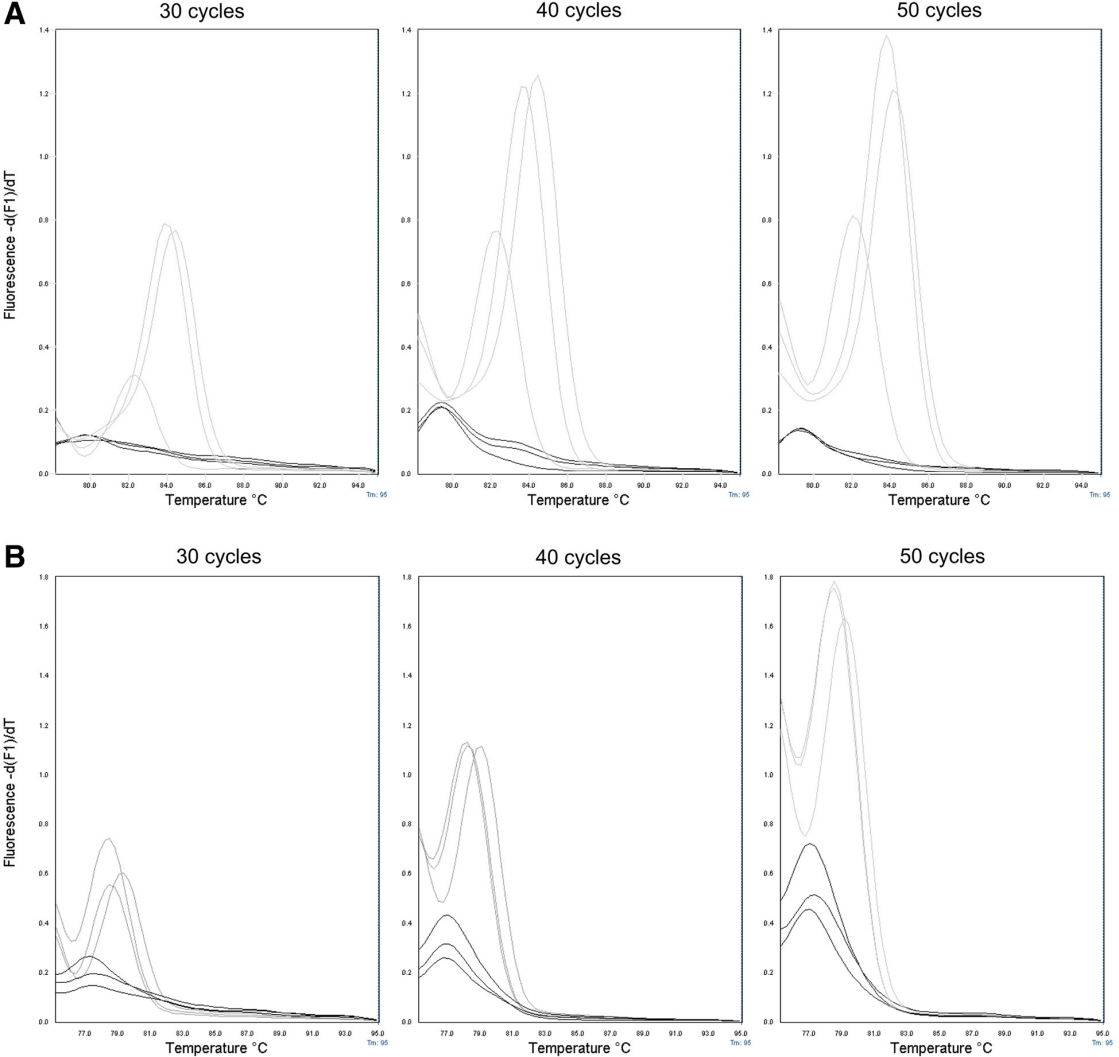
**Optimization of real-time PCR and MCA for the detection of B and T cell clonal rearrangement.** Samples of 3 dogs with B cell lymphoma (**A**, grey lines) and T cell lymphoma (**B**, grey lines) were amplified in parallel with regular lymphoid DNA from 3 dogs (black lines) for 30, 40 and 50 cycles. A total of 50 cycles for IgH major and of 40 cycles for TCRγ were found to be optimal for evaluation of clonal rearrangement, based on the peak height of clonal samples and the –d(F1)/dT ratio between lymphoma and non-neoplastic DNA. Peak height was measured for all samples from zero to the highest point of the melting curve.

Finally, a total of 50 cycles for IgH major and of 40 cycles for IgH minor and TCRγ was found to be optimal for the differentiation between lymphoma samples and non-neoplastic DNA based on the peak height of clonal samples and the peak height ratio (Figure 2). The overall peak height of non-neoplastic DNA was remarkably lower when amplified with the IgH major than with IgH minor (the latter not shown) and TCRγ primer sets. Moreover, the peak height of the regular lymphoid samples showed a much greater variability with TCRγ than with IgH major and minor. Differences of T_m_ between regular lymphoid and lymphoma DNA were greater with IgH major than with IgH minor and TCRγ primer sets.

Based on these settings, clonality of a sample was defined by a –d(F1)/dT ratio from sample DNA to regular lymphoid DNA of at least 1.5:1 for IgH major, 3:1 for IgH minor and 2:1 for TCRγ. In addition, the shape of the clonal peak had to be symmetrical. The peak height was measured for both clonal and regular lymphoid samples, from zero to the highest point of the melting curve to ensure consistent evaluation.

### Detection of clonal antigen receptor gene rearrangement in clinical cases

**Figure 3:**
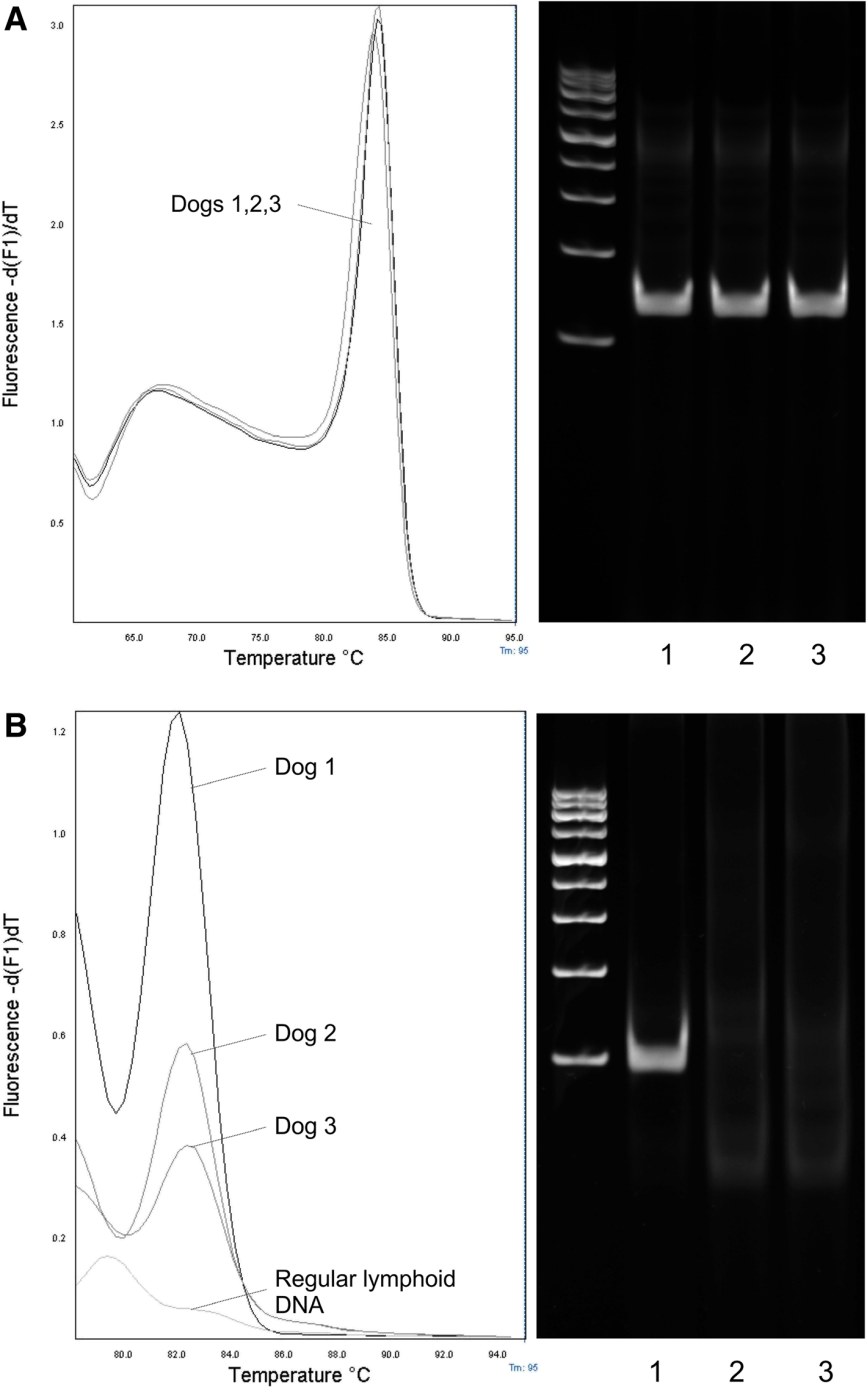
**Evaluation of 3 patients with B cell lymphoma by MCA and PAGE.** Samples of 3 dogs with B cell lymphoma were amplified with Cμ **(A)** and IgH major **(B)** primer sets by real-time and conventional PCR. Amplification with Cμ primers revealed comparable peak heights on MCA and distinct bands on PAGE for all dogs. Amplification with IgH major primers showed positive results for all 3 dogs on MCA, based on the cut-off of 1.5:1 of lymphoma to regular lymphoid DNA although peak heights varied significantly. Two of the dogs revealing lower peak heights on MCA (dogs 2,3) failed to show identifiable distinct bands on PAGE. All reactions were carried out with a total of 100 ng of DNA.

A total of 49 lymph node samples (40 from dogs with lymphoma, 9 from dogs with with lymph node hyperplasia) were analyzed by conventional and real-time PCR (Tables 2 and 3). Of the lymphoma samples, 30 were of the B cell type whereas 10 were T cell lymphomas as determined by flow cytometry. Internal control using Cμ indicated the presence of lymphoid tissue and sufficient DNA quality and integrity in all preparations by both PCR methods.With the conventional PCR system, the B cell immunophenotype was identified in 93% of samples of dogs with lymphoma (28/30: 23 IgH major, 2 IgH minor, 3 IgH major and minor) and the T cell type in 80% of samples (8/10). Most samples revealed positive results using a total amount of 100 ng of DNA whereas some required an increase of DNA to 500 ng per reaction. Samples of 4 dogs with lymphoma (2 with B and T cell lymphoma each) failed detection of clonal rearrangement with the increased DNA amount per reaction. Real-time PCR and MCA revealed B cell clonal rearrangement in 100% of lymphoma cases (25 IgH major, 2 IgH minor, 3 IgH major and minor) and the T cell type in 80% of cases (8/10). The T_m_ was 82.67°C ±1.22 for IgH major, 81.86°C ± 0.46 for IgH minor and 78.74°C ± 0.66 for TCRγ. Samples of 2 patients with T cell lymphoma revealed a large symmetrical double-peak with MCA. Gel electrophoresis of these samples showed 2 similar distinct bands for each sample, thus the corresponding samples were classified as clonal. The same 2 patients with T cell lymphoma that were missed with the conventional method failed to show clonal rearrangement with the real-time system. The 2 dogs with B cell lymphoma that were missed by conventional PCR and PAGE revealed smaller peak heights on MCA with IgH major than other samples (Figure 3B) although quality of lymphoid DNA was comparable (Figure 3A).

**Table 2:** Clonal antigen receptor rearrangements by conventional and real-time PCR in dogs with B cell lymphoma* (n = 30)

	**Conventional PCR**		
	**Rearranged**	**Not detected**	**Total**
Real time PCR			
Rearranged	28	2	30
Not detected	0	0	0
Total	28	2	30

*as determined by flow cytometry.

**Table 3:** Clonal antigen receptor rearrangement by conventional and real-time PCR in dogs with T cell lymphoma* (n = 10)

	**Conventional PCR**		
	**Rearranged**	**Not detected**	**Total**
Real time PCR			
Rearranged	8	0	8
Not detected	0	2	2
Total	8	2	10

*as determined by flow cytometry.

Samples that were identified by both PCR methods revealed clonal rearrangement of the same loci. None of the two PCR methods detected clonal rearrangement in the 9 samples with lymph node hyperplasia. With conventional PCR and PAGE, results remained negative even if increased amounts of DNA of 500 ng per reaction were used. With MCA, peak height and peak shape of hyperplastic samples was comparable to the polyclonal control (data not shown).

### Limit of detection of clonal antigen receptor gene rearrangement

The detection limit for clonal rearrangement was determined for conventional and real-time PCR using dilutional series of lymphoma samples with water and with regular lymphoid DNA.

Logarithmic dilutions into water ranging from 100 ng to 0.01 ng were prepared of 6 samples each with IgH major, minor and TCRγ clonality. Analysis of the dilutional series by conventional PCR and PAGE revealed visible bands to a concentration of 0.3 ng (median 10 ng) with IgH major, to 10 ng (median 30 ng) with IgH minor and to 3 ng (median 10 ng) with TCRγ. With real-time PCR and MCA, clonal rearrangement was demonstrated to 0.1 ng (median 0.1 ng), 1 ng (median 3 ng) and 0.3 ng (median 1 ng) with IgH major, minor and TCRγ.

**Figure 4:**
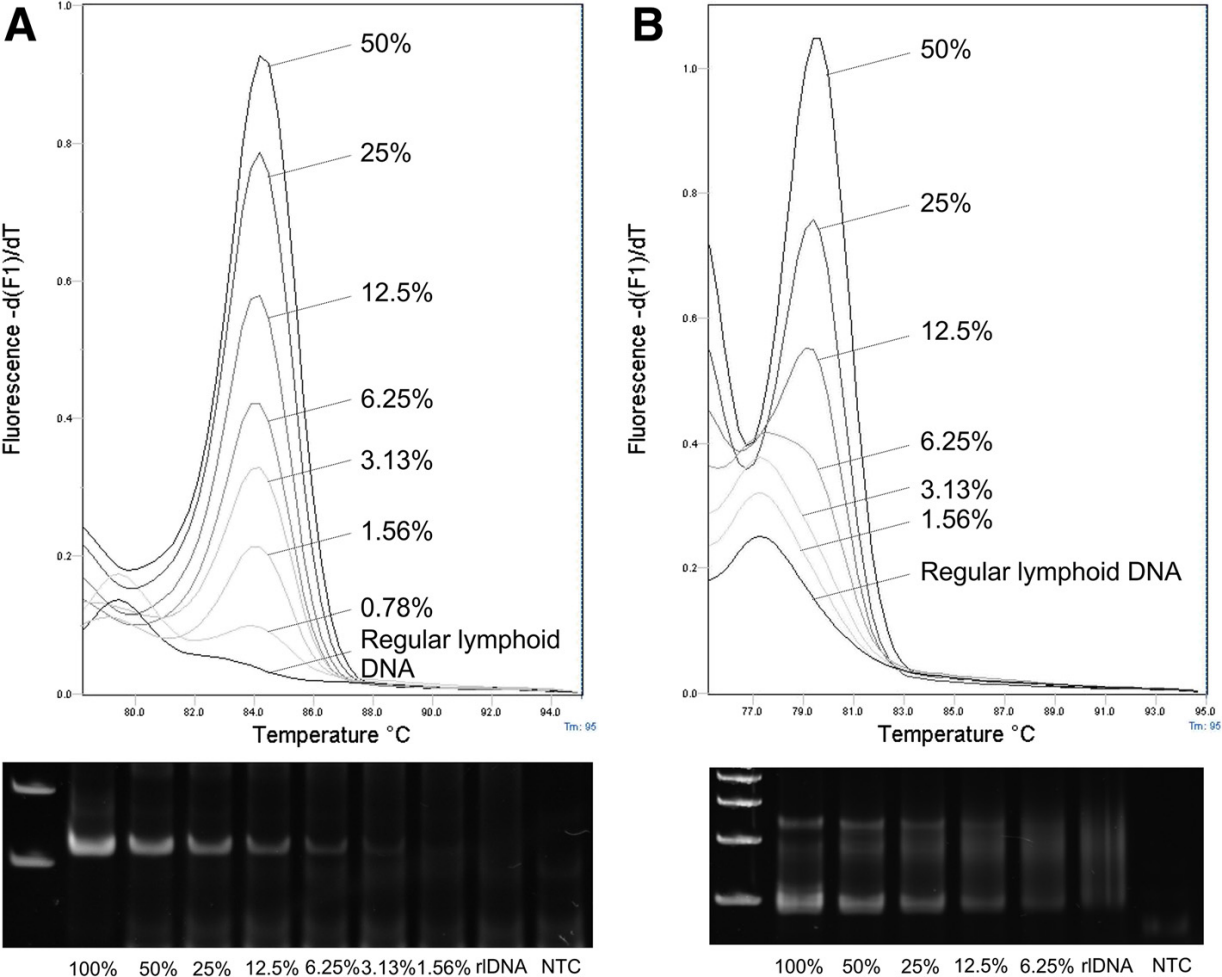
**Dilutional series of lymphoma DNA analyzed by conventional and real-time PCR.** Lymphoma samples of a dog with B cell lymphoma **(A)** and T cell lymphoma **(B)** were serially diluted into regular lymphoid DNA (rlDNA; 50%, 25%, 12.5%, 6.25%, 3.13%, 1.56%, 0.78%) and amplified with IgH major and TCRγ primer sets. The minimal concentration producing a visible band was 3.13% and 12.5% for B and T cell clonal rearrangement with conventional PCR and subsequent PAGE. The detection limit for real-time PCR with MCA was 1.5% and 6.25% for B and T cell clonal rearrangement, based on the cut-off of a –d(F1)/dT ratio from sample DNA to regular lymphoid DNA of at least 1.5:1 for IgH major and 2:1 for TCRγ. The regular lymphoid DNA was prepared as a mix of 12 lymph node samples of 3 dogs to adjust for peak height variations between individual samples as observed for the amplification of regular lymphoid DNA with the TCRγ primer set (Figure 2B). Gel images include one lane with 100% lymphoma DNA and a non-template control (NTC).

**Figure 5:**
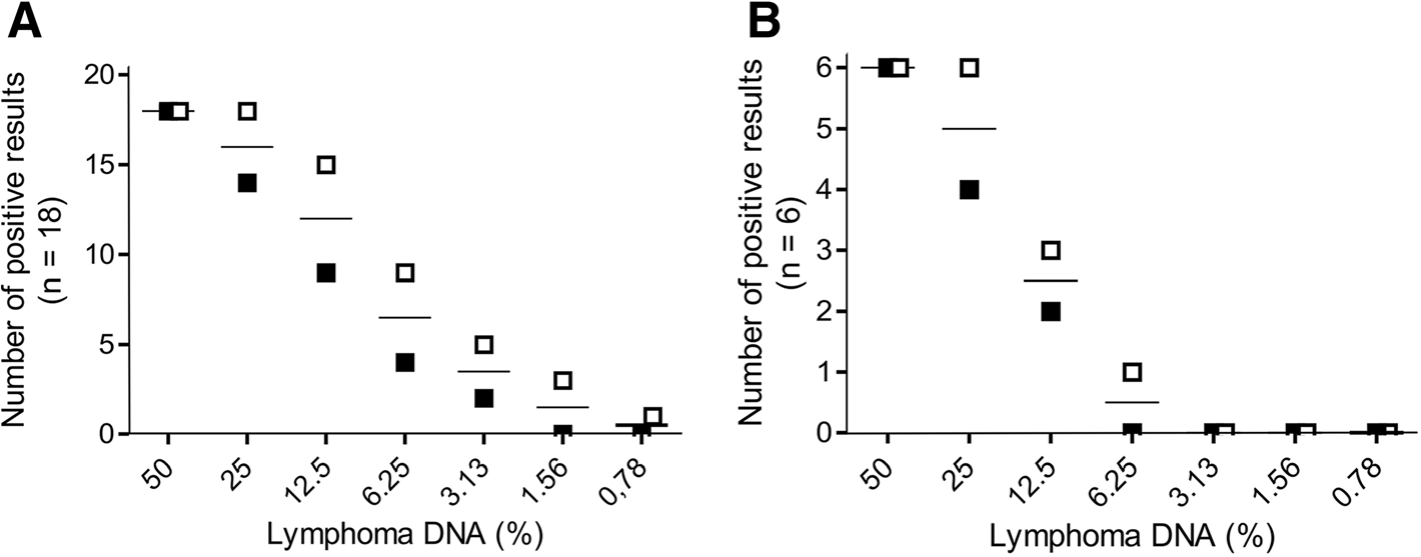
**Comparison of detection limits between conventional and real-time PCR.** Serial dilutions of DNA samples of 18 dogs with B cell lymphoma **(A)** and 6 dogs with T cell lymphoma **(B)** were prepared with regular lymphoid DNA (50%, 25%, 12.5%, 6.25%, 3.13%, 1.56%, 0.78%) and amplified with IgH major and TCRγ primer sets by both PCR methods. The number of positive results obtained with PAGE (black squares) and MCA (white squares) is indicated for each concentration.

To evaluate the detection limit for clonal rearrangement in the presence of non-neoplastic lymphoid tissue, 2-fold dilutional series (50% to ~0.4% of lymphoma DNA) were prepared of 12 samples with IgH major clonality and of 6 samples each of IgH minor and TCRγ clonality (Figure 4). Conventional PCR showed positive results to 3.13% (median 12.5%) of lymphoma DNA with IgH major and minor and to 12.5% (median 25%) with TCRγ. Real-time PCR revealed detection of clonal rearrangement to 0.78% (median 9.38%), 3.13% (median 12.5%) and 6.25% (median 18.5%) of neoplastic DNA with IgH major, IgH minor and TCRγ, respectively. The total number of samples showing clonal rearrangement at the lower concentrations of lymphoma DNA was higher for real-time PCR than for the conventional method (Figure 5).

## Discussion

Real-time PCR with MCA is used in various human lymphoid neoplasias for the diagnosis of clonal antigen receptor gene rearrangement by its characteristic pattern on MCA [13,14,19-22,24-26]. The method enables a reliable differentiation between neoplastic and regular lymphoid tissue with a minimum of hands-on time. The present study describes the development and evaluation of a real-time PCR with MCA for the detection of canine lymphoma using previously established primer sets [6]. The method was assessed in comparison with a conventional PCR system, utilizing a total of 40 lymph node aspirates from dogs with B or T cell lymphoma and a total of 9 samples of dogs with lymph node hyperplasia.The real-time PCR system used in the present study revealed a similar pattern on MCA for lymphoma samples as described for human lymphoproliferative neoplasias. Based on the tall and symmetrical shape of the peaks, all samples with the exception of 2 dogs with T cell lymphoma showing flat and irregular peaks, would have been correctly identified. Some studies in human medicine exclusively utilized the presence of a symmetrical peak for diagnosing clonal rearrangement [13,14,19]. However, this method is difficult to standardize. In the present study we used an optimized ratio of the peak height between lymphoma and regular lymphoid DNA as an additional criterion to the peak shape, as previously described for human T and B cell neoplasias [20-22,26]. Since the peak height not only relies on the clonality but also on the amount of DNA in the sample, the use of identical amounts of DNA in both sample and regular lymphoid control is warranted to obtain reliable results. Moreover, although real-time PCR system as described in the present study enables facilitated interpretation of clonality results to some degree, it may become necessary in certain applications (e.g. high polyclonal backgrounds in MRD) to adjust the cut-offs accordingly.

The real-time PCR and MCA revealed comparable sensitivity and specificity to the conventional PCR system with PAGE. The results are in concordance with most studies of human lymphoid neoplasia that revealed relative sensitivities of 100% and specificities of 97% to 100% for MCA compared to PAGE [20,22]. In the present study, 2 dogs with B cell lymphoma detected by the real-time PCR system were missed by conventional PCR with PAGE (Figure 3). The low peak height on MCA following the amplification with IgH major compared to other samples may be suggestive of a reduced amount of clonally rearranged DNA in these samples. According to the peak heights seen for Cμ, it can be assumed that the quality of lymphoid DNA was comparable to other samples. It may be concluded, that real-time PCR with MCA was more capable of detecting small amounts of clonally rearranged DNA than conventional PCR with PAGE. However, as capillary electrophoresis has been proven superior to PAGE for the resolution of clonally rearranged DNA in dogs with lymphoma [11,12], the use of this method may have improved the sensitivity of the conventional system.

Comparison of MCA to PAGE in human B and T cell malignancies showed an improved detection limit for dilutions of lymphoid DNA [13,20,22,26]. Overall detection limits for MCA ranged between 0.25% and 12.5% of neoplastic DNA for B cell clonality, and 6.25% to 10% for T cell clonality. The limits were up to 4-fold higher than for conventional PCR [26]. In the present study, the lower detection limits for IgH major and TCRγ were 4- and 2-fold higher for MCA compared to PAGE, thus indicating an increased analytical sensitivity of the real-time PCR. However, as above, the use of conventional PCR with capillary electrophoresis as [11,12] may have improved the detection limit in the present setting.

Real-time PCR with MCA failed to detect clonal rearrangement in samples of 2 patients with T cell lymphoma. The poor performance of PCR in the current setting is likely due to the primer set used for the amplification of the canine TCRγ [27,28]. Simplex primer sets as used in the present study can miss clonality in a multicassette structure like the canine TCRγ. This assumption was supported by the failure of conventional PCR and PAGE to detect clonal rearrangement in the same 2 patients. A recently developed multiplex PCR revealed a markedly improved sensitivity for the detection of TCRγ rearrangement compared to single primer sets [12] and may also be applicable to real-time PCR with MCA. A less likely explanation for the failure of detection in these 2 cases may be the aspiration of lymphoid tissue other than the neoplastic population. This cannot be ruled out in the present study as the sampling process involved one aspirate that was analyzed by cytology and flow-cytometry and a second aspirate that was used for DNA extraction and subsequent amplification without prior microscopical assessment.

Detection limits of both PCR methods were inferior for T cell clonality compared to samples of dogs with B cell lymphoma. Similar findings have been described previously in veterinary medicine [6]. Conventional PCR and PAGE in a dog with B cell leukemia revealed clonal rearrangement at concentrations as low as 1%, whereas a dog with T cell neoplasia had a detection limit of 10% of neoplastic DNA. Recently, detection limits of 5% using a multiplex PCR and 1% using simplex assays were described [12]. Detection of clonal products was partially hampered by the polyclonal background caused by the multicassette structure of TCRγ. Similarly, real-time PCR with MCA revealed a markedly elevated polyclonal background for TCRγ compared to IgH major and minor (Figure 2A,B). In addition, differences in T_m_ between regular lymphoid DNA and lymphoma DNA were smaller for the amplification with TCRγ than for IgH major and minor. Both findings likely contributed to the reduced detection limit of the real-time PCR system for T cell lymphoma in the present study.

One limitation of the real-time PCR is the lack of standardization of B and T cell DNA in samples of dogs with and without lymphoma. A reliable comparison of peak heights ideally requires similar amounts of either B or T cell DNA in sample and control as a shift towards one population may result in an inflation of the corresponding melting peak. To avoid false positive results, peak shape was established as a second criterion for diagnosing clonal rearrangement. Thus, non-neoplastic samples containing a higher amount of B or T cell DNA than the control are likely to be identified as polyclonal by the presence of a rather wide and potentially asymmetrical peak.

A recent study investigating MRD in dogs with B cell lymphoma revealed detection limits of 1 lymphoma cell per 10.000 non-neoplastic lymphoid cells [9]. Individually designed primers, standard curves and probes targeting the CDR3 region were utilized for the evaluation. By contrast, in the present study, universal primers were used that amplify both, neoplastic and regular lymphoid DNA, thereby reducing the overall sensitivity of the method for the detection of clonal rearrangement. This is especially true when small amounts of tumor cells and large amounts of non-neoplastic lymphoid cells are expected as in MRD. However, MRD was detected with universal primers in the peripheral blood of dogs with lymphoma that were in complete remission using conventional PCR with PAGE [29]. Therefore, detection of MRD by real-time PCR and MCA in combination with universal primers may be possible.

## Conclusion

In the present study, it was demonstrated that real-time PCR with MCA is a sensitive and specific method for the detection of clonally rearranged DNA on non formalin-fixed and paraffin-embedded lymph node samples. The real-time PCR system combines amplification reaction and subsequent assessment of clonal rearrangement by MCA in the same instrument and reaction vessel. This eliminates manual post-PCR processing of the product and makes the method less labour- and time-intensive than conventional PCR with PAGE. Costs of materials for the real-time PCR are comparable those for the conventional system. Thus, the real-time PCR method with MCA has the potential for assisting the diagnosis of antigen receptor gene rearrangement in canine lymphoma patients in a clinical setting.

## Abbreviations

CDR3: Third complementarity determining region; Cμ: Constant region gene of immunoglobulin M; -d(F1)/dT: Peak height melting curve obtained by plotting the negative first derivative of the fluorescence (F) versus the temperature (T); DNA: Desoxyribonucleic acid; IgH: Immunoglobulin heavy chain; MCA: Melting curve analysis; MRD: Minimal residual disease; PCR: Polymerase chain reaction; TCRγ: T cell receptor γ; Tm: Melting temperature; PAGE: Polyacrylamide gel electrophoresis.

## Competing interests

The authors declare that they have no competing interests.

## Authors’ contributions

KFAL conceived and designed the study, carried out the molecular methods, analyzed the data and drafted the article. AEJ and VE participated in the coordination and management of the study and provided biological specimen. HJS and MK made substantial contribution to the molecular analysis of specimen. NE, IN and DB participated in the study design and edition of article. All authors read and approved the final manuscript.
